# Recommendations from primary care providers for integrating mental health in a primary care system in rural Nepal

**DOI:** 10.1186/s12913-016-1768-9

**Published:** 2016-09-19

**Authors:** Bibhav Acharya, Jasmine Tenpa, Poshan Thapa, Bikash Gauchan, David Citrin, Maria Ekstrand

**Affiliations:** 1 Possible, Bayalpata Hospital, Sanfebagar-10, Achham, Nepal; 2 Department of Psychiatry, University of California, 401 Parnassus Ave, Langley Porter, San Francisco, 94143 CA USA; 3 Shared Minds, Boston, MA USA; 4 Department of Anthropology, University of Washington, Seattle, WA USA; 5 Department of Global Health, University of Washington, Seattle, WA USA; 6 Henry M. Jackson School of International Studies, University of Washington, Seattle, WA USA; 7 Department of Medicine, University of California, CA San Francisco, USA

**Keywords:** Mental health, Global health, Nepal, Task-shifting, Health systems strengthening, Implementation research, Focus group discussions

## Abstract

**Background:**

Globally, access to mental healthcare is often lacking in rural, low-resource settings. Mental healthcare services integration in primary care settings is a key intervention to address this gap. A common strategy includes embedding mental healthcare workers on-site, and receiving consultation from an off-site psychiatrist. Primary care provider perspectives are important for successful program implementation.

**Methods:**

We conducted three focus groups with all 24 primary care providers at a district-level hospital in rural Nepal. We asked participants about their concerns and recommendations for an integrated mental healthcare delivery program. They were also asked about current practices in seeking referral for patients with mental illness. We collected data using structured notes and analyzed the data by template coding to develop themes around concerns and recommendations for an integrated program.

**Results:**

Participants noted that the current referral system included sending patients to the nearest psychiatrist who is 14 h away. Participants did not think this was effective, and stated that integrating mental health into the existing primary care setting would be ideal. Their major concerns about a proposed program included workplace hierarchies between mental healthcare workers and other clinicians, impact of staff turnover on patients, reliability of an off-site consultant psychiatrist, and ability of on-site primary care providers to screen patients and follow recommendations from an off-site psychiatrist. Their suggestions included training a few existing primary care providers as dedicated mental healthcare workers, recruiting both senior and junior mental healthcare workers to ensure retention, recruiting academic psychiatrists for reliability, and training all primary care providers to appropriately screen for mental illness and follow recommendations from the psychiatrist.

**Conclusions:**

Primary care providers in rural Nepal reported the failure of the current system of referral, which includes sending patients to a distant city. They welcomed integrating mental healthcare into the primary care system, and reported several concerns and recommendations to increase the likelihood of successful implementation of such a program.

**Electronic supplementary material:**

The online version of this article (doi:10.1186/s12913-016-1768-9) contains supplementary material, which is available to authorized users.

## Background

Worldwide, mental illness is the largest contributor to disability-adjusted life years (DALYs) from chronic illnesses [1]. Yet in many low- and middle-income countries (LMICs), there is often only one psychiatrist or psychologist for over two million people [2]. Estimates suggest that an additional 1.2 million providers are needed to meet mental health care needs [3]. Task-sharing (also referred to as “task-shifting”), the involvement of non-specialist service providers to deliver mental health services, has received much attention as a key aspect of closing this gap and scaling up healthcare services [4–6]. Task-sharing in LMICs often includes training Primary Care Providers (PCPs) to diagnose and manage common mental illnesses under the supervision of a psychiatrist [7, 8]. A Cochrane Collaboration Review of such models showed improved outcomes for both mental health (e.g. rates of remission of depression) and physical health (e.g. diabetes care) in populations that otherwise lack direct access to mental healthcare specialists like psychiatrists and psychologists [9]. Similar studies have been conducted in some LMICs and have been shown to decrease rates of depressive and anxiety disorders [10].

Although such integrated programs hold much promise for LMICs, success largely rests on the PCPs. To ensure effective implementation of such programs, it is important to understand the current system in place for mental healthcare services, and to hear PCPs’ reactions to mental health service integration.

To assess these issues, we conducted three focus groups at a district-level hospital in rural Nepal. There are 54 psychiatrists in Nepal and most of them are located in Kathmandu [11, 12]. However, over 80 % of the country’s population live in rural regions [13]. Mental healthcare receives 0.7 % of the national health budget, most of which is spent on a stand-alone mental hospital in the capital [14]. The nearest psychiatrist is 14 h by road from Achham, our study site.

Achham is one of the poorest districts in Nepal, and was severely affected by the 10-year Maoist War that ended in 2006 [15]. Although prevalence data are not available for Achham, studies in other regions of Nepal have found rates of depression between 17–43 and that for PTSD between 8–14 % [16, 17]. Kathmandu is a 30 h bus ride away, and the nearest commercial airport is 10 h away. Since 2008, *Possible*, a non-profit health care organization, has been operating a district-level hospital in Achham in partnership with the Nepali government. The 25-bed general hospital employs over 150 staff and has seen more than 300,000 patients since 2008 [18]. The outpatient primary care clinic serves about 200 patients a day using an urgent care model, and is staffed by 12–15 PCPs at any one time. We conducted the focus groups to inform the development and implementation of an integrated mental healthcare delivery program at the hospital.

## Methods

### Participants

Any clinician engaged in directly assessing and treating patients in the outpatient primary care clinic was defined as a PCP. This included auxiliary health workers (AHWs), who completed 15–18 months of undergraduate training including 3 months of clinical rotations; health assistants (HAs), who completed 36 months of undergraduate training including 6 months of clinical rotations; and Bachelor of Medicine, Bachelor of Surgery (MBBS) physicians, who completed 5 years of undergraduate training including a 1-year clinical internship. All PCPs (*n* = 24) present in the hospital participated in the focus groups. AHWs, HAs, and MBBS physicians participated in separate focus groups to minimize the impact of workplace hierarchy on the discussions.

### Study Design

We conducted three focus groups, each lasting 60 min, and included all the PCPs in the hospital. Guiding questions (see Additional file 1) included some general and probing questions on specific elements of an integrated care system, such as an off-site consultant psychiatrist and an on-site mental health worker [19].

BA conducted the focus groups in Nepali and took structured notes in English for physicians and HAs, and in Nepali for AHWs. We shared these notes with the participants to maintain integrity of the data [20]. We considered notes final after all participants agreed that their perspectives had been adequately captured. BA translated the Nepali structured notes before coding. BA recorded all participant quotes in Nepali and translated into English.

We analyzed the data using a template approach of thematic analysis [21] utilizing an iterative process, resulting in a codebook with hierarchies and themes as described in Table 1. Four co-authors (BA, JT, PT and DC) participated in this iterative process and any disagreements were resolved by discussion among the coders until consensus was achieved. All four have received training in qualitative research methods.

**Table 1:** Summary of participants’ concerns and recommendations on integrating mental health services into primary care

Concerns	Recommendations
Integration of counselors into the primary care clinic	
1. Workplace hierarchies	1. Train current PCPs as counselor^a^
2. Lack of true collaboration between counselor and PCP	2. Co-manage patients between PCPs and counselors
3. Current clinic space may not provide privacy for counselor encounters	3. Create private space for mental health evaluations
4. Staff turnover and continuity of care	4. Recruit a senior and a junior counselor. If the senior person cannot be retained, the junior counselor will have received mentorship. 5. Use manualized therapy so a new counselor can take over care using the same principles of treatment.
5. High patient load for counselor	6. Consider group therapy rather than one-on-one therapy.
Consultation from an off-site psychiatrist	
6. Reliability of off-site psychiatrist	7. Prioritize recruiting academic psychiatrists, who may have a flexible schedule and be reliable.
7. High number of patients for case review	8. Discuss amongst on-site clinicians first to decide which patients to discuss 9. Develop a priority order (e.g. by severity) and discuss those patients first, rather than trying to discuss all patients.
8. Consultation questions may not wait until the weekly review meeting	10. Allow urgent consultation throughout the week, in coordination with the PCP and counselor.
Training and Support for PCPs	
9. PCPs may not be able to appropriately screen patients for mental illness	11. Train and support PCPs in screening, diagnosis and treatment of mental illness 12. Integrate screening tools into the medical records system
10. PCPs may not have the requisite clinical skills to follow the psychiatrist’s recommendations	13. Provide on-site training on clinical skills by a visiting psychiatrist
11. Risk of abuse of psychiatric medications.	14. (No recommendation)

^a^Counselor: Psychosocial Counselor

## Results

6 MBBS physicians, 11 HAs, and 7 AHWs participated in three focus groups. We organized the results into major themes with representative quotes, and any notable differences among the three groups have been highlighted. Participant’s reactions to an integrated mental health delivery program are summarized in Table 1.

### Current referral practices for patients with mental illness

AHWs and HAs noted that they first sought supervision from MBBS physicians in their own clinic. In addition, they may have internally referred patients to one HA who had received 4 weeks of mental health training. Although this was considered better than having no one with mental health training, they noted that the one HA would often be overburdened, particularly because patients needed extra time for mental health evaluation. After exhausting these options of internal referral, all participants noted that they would ask patients to travel to the city to see a psychiatrist, the nearest one being 14 h each way by bus.

Everyone thought this was a major burden on the patients. Very few patients could afford the travel and associated costs of visiting the psychiatrist in the city. However, families would often procure large, high-interest loans that are several times the average annual income in the region, and travel to India or Kathmandu for a psychiatric consultation. Several participants noted that many patients returned with inappropriate medications (e.g., benzodiazepine monotherapy for depression) and tests, and no plan for follow-up:

*“A patient went to Kathmandu. They went to several hospitals and did many head CT scans. They spent so much money but were tricked. It was all normal. They needed mental health care.”*

*“Many patients go to India, spend a lot of borrowed money and they have to go back to India for follow-up. It is very expensive for them.”*

All participants desired a more integrated approach to mental healthcare services in their own practice setting.

### Integration of psychosocial counselors into the primary care clinic

Psychosocial counselors are a specific cadre of mental healthcare workers in Nepal. After graduating from high school, they receive a 6 month classroom-based and workplace-based, mentored training on understanding mental disorders, coordinating care, teaching relaxation and other stress-reduction techniques, and providing psychoeducation and basic psychosocial support to patients and families [22].

Participants had several concerns and recommendations about integrating an on-site counselor into the primary care clinic. They were concerned about the perception of counselors as being seen as “lower” than the PCP, primarily because the former would not be a prescriber, a status that comes with much respect in the healthcare delivery system. They were concerned that PCPs and counselors would thus have difficulty collaboratively working as part of a team. All participants made the recommendation that instead of hiring counselors, existing HAs or AHWs should be sent to receive training to become a counselor. This would merge the two categories and also result in counselors who understand the existing healthcare system well and can quickly integrate into the primary care clinic:

*“Instead of trying to hire someone from outside, it might be better to train our own staff. I am ready to go, and I am sure my colleagues are, too. We know our system and it will be easier for us to come back and work with our colleagues.*”

An additional concern about counselor integration related to the potential problem of patient “dumping”. Participants cautioned against creating an insulated counselling clinic that could result in PCPs feeling like they do not have to take care of the referred patients anymore, and the counselors feeling like the patients would no longer be evaluated by the PCPs. This could result in poor, fragmented care even if the PCPs and counselors are co-located. Participants recommended that it should be made clear to all stakeholders that the patients will be continued to be seen by both PCPs and counselors, can be referred back and forth at any time, and that all providers will collaborate to develop appropriate treatment plans:

*“If we refer the patient to a counselor and think, “Ok, now I don’t have to think about this patient”, then that is not good. We should all work together for the patient. The patient should also go back to us if the counselor wants to refer the patient to us.”*

The clinical space in the primary care clinic did not allow confidentiality for patients, as PCPs share offices. Patients and family members could easily overhear other patients during an evaluation. Participants were concerned that this structure would not provide the kind of private space needed to discuss sensitive topics about mental health, substance use, and social problems. They recommended that a separate space should be created where counselors could engage in confidential conversation with the patients.

Since staff turnover is a major challenge in low-resource, rural settings, participants were concerned that continuity of care for patients may suffer. This was seen as a particularly grave concern given the importance of the connection between patients and a mental health service provider (versus a generalist PCP). Participants recommended that a senior and junior counselor should be recruited and both should engage in providing care to the same patients:

*“Senior counselors will not stay for long in remote regions. If we hire both senior and junior counselors, even if the senior person leaves, the junior one will continue to provide care.”*

Several participants who were familiar with psychotherapy suggested using protocol-based, manualized therapy techniques so that a new counselor would know what was being done with the patients, and continue treatment where the previous counselor had left off. Participants also noted that training existing PCPs as counselors could help with staff retention.

The final concern regarding integration of counselors related to the high volume of patients they may encounter. Given that over 200 patients are seen in the outpatient clinic, counselors may quickly fill up their slots. A couple of participants, who were aware of various psychotherapeutic techniques, suggested the following:

*“If counselors spend 1 h with each patient, we will not be able to refer more patients to them. They should do psychosocial counseling with many patients at the same time.”*

### Consultation from an off-site psychiatrist

Participants were asked for their thoughts on involvement of an off-site consultant psychiatrist, who would conduct a case review of a panel of patients with mental illness. Compared to a model that relies upon on-demand consultation requests by the PCPs, panel review allows psychiatrists to address blind spots of PCPs and consult on a significantly larger number of patients [19, 23]. All participants felt positively about involving a specialist in mental healthcare services. They were concerned that since the psychiatrist is not an on-site, full-time employee, he or she may not be able to consistently set aside time every week to conduct panel reviews:

*“I think bringing specialist into our system will help our patients a lot. But if the specialist is in Kathmandu and is working part-time, will they be committed and reliable?”*

All MBBS physicians agreed when one of the members of their focus group suggested recruiting psychiatrists based at an academic medical center in Nepal. Participants noted that such psychiatrists are likely to be more reliable with their schedule compared to someone who is in private practice, and has more lucrative competing interests.

After hearing about the model where all patients with mental illness are included in the panel, participants were concerned that it would be very difficult to discuss all the patients during the panel review. All participants suggested that it would be better to have a way to sort the panel by severity and prioritize some patients:

*“Maybe we should only talk about patients who are severely ill and complicated.”*

*“As HAs, we can get together and then discuss patients first. Then we can decide who should be presented to the psychiatrist”*

The final concern about psychiatric consultation was regarding urgent calls. Participants acknowledged the importance of a panel review and the challenge the psychiatrist faced in answering calls throughout the week. Yet, they also described situations where they would not be able to wait until the weekly consultation:

*“If a patient is very sick, we will have to call the psychiatrist for urgent consultation.”*

To avoid having more than 15 PCPs with varying levels of prior training and comfort in mental healthcare directly call the psychiatrist, the recommendation was to have PCPs discuss cases with counselors, and then mutually decide if an urgent call to the psychiatrist was warranted:

*“If we discuss with the counselor first, all the questions will come from the same counselor, which is good for the psychiatrist. The counselor may even be able to answer some of those questions, so we don’t have to call the psychiatrist all the time.”*

### Training and Support for PCPs

All participants recognized their critical role in the integrated mental healthcare delivery program:

*“This program will not be successful if we do not screen patients. The psychiatrist and counselor will never receive an opportunity to help the patient.”*

Given the varying level of mental healthcare training and comfort among the PCPs [24], they emphasized the importance of training and support in appropriate screening, diagnosis, and treatment of mental illness. Some participants suggested using posters, manuals, and books as reference materials during the clinical encounter. However, when asked how often such resources were actually used, participants noted that they are often impractical in a busy clinical setting where pausing a visit to refer to protocols is not feasible. Given that this hospital has an electronic health record (EHR) system, participants suggested including screening tools, diagnostic criteria, and treatment protocols directly into the medical record platform:

*“Using the EHR system will be better. That way, we can quickly look at the protocol when we are still seeing patients. We won’t have to look for a book or go to a different room.”*

Participants were also concerned about their ability to comprehend and implement the recommendations from the off-site psychiatrist. This was driven by lack of familiarity with using specific interviewing techniques, clarifying confusing diagnoses, and providing brief counseling. Participants noted that reference materials, training lectures, and clinical protocols would not address this gap. They recommended on-site, hands-on training:

*“The psychiatrist may ask us or the counselor to do something, and we may not know how to follow those directions. If the psychiatrist can come here and train us, we will know how to appropriately follow the recommendations.”*

One MBBS physician expressed concern about increasing access to psychotropic medications:

*“I think we have to be careful about writing a lot of prescriptions for psychiatric medications, especially by non-physicians or those who have not received appropriate training. Patients can become addicted to some medications.”*

Participants made no specific recommendations to address this concern.

## Discussion

The insights from the focus groups can inform the process of implementing integration of mental healthcare services into primary care in rural Nepal. The challenges in obtaining mental healthcare referral are typical of low-resource settings. PCPs’ recognition of the failure of the current system to improve patients’ conditions may have made them welcoming of an integrated mental healthcare system. This is comparable to the findings from a multi-country study on task-sharing [25]. Our study found that concern about workplace hierarchy between PCPs and counselors is a critical challenge in successful collaboration. Although participants note that this is driven by the perceived lower status of non-prescriber healthcare workers, it is also possible that counselors face additional stigma as *mental* healthcare workers [26]. Given that the focus group facilitator is a mental healthcare worker, this issue may not have been raised by participants. Participants recommend sending current PCPs for training as counselors but this may not always be feasible or desirable. Considering that the length of the training for counselors, this could mean delaying the mental healthcare program by at least 6 months. Recruiting existing counselors rather than waiting for current staff to be trained would expedite implementation, and would require other strategies to minimize hierarchies: recruiting clinic leadership to introduce non-prescribing counselors as an integral part of the team, and having counselors provide some of the mental healthcare training that the PCPs are seeking.

Participants’ recognition that the lack of mental healthcare training among PCPs can severely affect the program presents an important opportunity. Their desire for training can be addressed by developing a program that includes didactic teaching about mental health, provision of validated screening tools [27], and skills-building training on patient-provider communication, assessment, and diagnosis. Their concern about their own skills in providing mental healthcare services can be address by training and utilizing a rating scale that has been validated in Nepal [28].

The only concern that did not present with a specific recommendation from the participants was regarding the abuse of psychiatric medications. This can be addressed with extensive de-prioritization of benzodiazepines during mental healthcare training. Adding safeguards in the clinic, such as limiting prescriptions to few PCPs, providing only a short course of medications, and frequently reassessing the need for continued renewals may also address this concern. In addition, it will be important to address the myth that all psychotropic medications cause dependence. Compared with another study that looked at PCPs’ response to task-sharing, we did not find that participants were concerned about the increased burden in providing mental health services. This could have been because of the central role of counselors, who can relieve busy PCPs [11, 29]. Also notably absent from the discussions is the availability of psychotropic medications. Given this hospital’s close partnership with the Ministry of Health, which provides a regular supply of psychotropic medications, and this hospital’s inventory tracking system, medication stockouts may not be as common here as in other LMIC settings.

One limitation of this study is that participants were asked to imagine how they would integrate mental healthcare services, but were presented with two specific strategies: integrating counselors in the clinic and case review with an off-site psychiatrist. A truly open approach that asked them about various strategies on integration may result in more diverse insights. Given that the focus group leader is a psychiatrist, we were also concerned about potential bias among participants to avoid criticism of the mental health program. To encourage critical views, question 5 and 6 explicitly asked for PCPs’ concerns.

Other limitations relate to the applicability of these findings in other settings. About a year before this study, some of the participants had attended two pilot lectures on screening for depression and psychosis. This may have made some of them more aware of mental health issues, as evidenced by their acknowledgment of the importance of psychotherapy and specific suggestions on reducing the patient load for counselors. This may have led to a more favorable and welcoming approach to integration of mental healthcare services. In other settings, PCPs may have a vastly different conceptualization of mental illness and may not acknowledge the importance of providing mental health services.

One key challenge in adapting integrated mental healthcare in LMICs is that the outpatient clinic may often have an urgent care model rather than a strictly primary care model. Patients do not have a specific appointment with a specific clinician, but rather line up in the morning and are seen sequentially by whichever clinician has an opening. The participants’ implicit awareness of this challenge may have resulted in their relative openness to integrate counselors, who would provide continuity of care for patients even if different PCPs see the same patient in the clinic. It is possible that in a different setting, where a traditional primary care model is utilized, PCPs will report a different set of challenges and strategies in collaborating with a counselor.

Future studies are needed to elicit similar perspectives from counselors, psychiatrists, and patients about the integration of mental healthcare services into existing primary care delivery. Finally, the results from this study can inform implementation science studies on integration of mental healthcare into primary care. Various indicators, some of which have been identified by a Delphi study that included Nepal [30], may be used to track the process and impact of such a program: changes in number of patients referred to the city; perceived hierarchies between counselors and PCPs; availability of a private space for confidential patient encounters; counselor turnover rate; average length of time spent on the waitlist to see a counselor; perceived reliability of the off-site psychiatrist; attendance rate of off-site psychiatrist during panel review meetings; number of patients discussed during panel review; number of times psychiatrists are called outside of the designated panel review; and the impact of training on self-efficacy among PCPs in screening patients and then following recommendations made by the psychiatrist.

## Conclusions

PCPs recognize that current referral practices pose a large burden on patients. PCPs also have a positive view of integrating mental healthcare services into their primary care setting. They provide several concerns and recommendations about recruiting an embedded psychosocial counselor and an off-site psychiatrist. They seek additional training in mental health, and are acutely aware of systems-wide issues that may affect the success of the program. Similar qualitative studies have the potential to inform implementation of mental health programs in low-resource primary care settings in other regions of the world.

## Additional file

Additional file 1: Title of data: Guiding questions for the focus group discussions. Description of data: List of questions used to guide the focus group discussions. (DOCX 14 kb)

## Abbreviations

DALY: Disability-adjusted life year
LMIC: Low- and middle-income country
PCP: Primary care provider
AHW: Auxiliary health worker
HA: Health assistant
MBBS: Bachelor of Medicine, Bachelor of Surgery
EHR: Electronic health record
