# Supplementary material for: Recommendations from primary care providers for integrating mental health in a primary care system in rural Nepal
Source: BMC Health Serv Res. 2016 Sep 19;16:492. doi: 10.1186/s12913-016-1768-9 (PMC5028958; doi:10.1186/s12913-016-1768-9)
Supplement: Additional file 1: — Title of data: Guiding questions for the focus group discussions. Description of data: List of questions used to guide the focus group discussions. (DOCX 14 kb) [file 12913_2016_1768_MOESM1_ESM.docx]

Guiding questions for the Focus Group Discussions:

1. What do you currently do with patients who have a mental illness and need a referral?
2. How does this referral process affect the patients?
3. What are your thoughts on receiving online/phone-based consultation from a consultant psychiatrist based off-site in the city?
4. What are your thoughts on mental healthcare workers integrated into your clinic with the primary tasks of providing counseling to patients, and coordinating care by relaying clinical information between you, the consultant psychiatrist and the patient?
5. What concerns do you have about a program that adds this new structure to your current practice?
6. What are your recommendations to address those concerns?
